# Female travellers in hospitality and tourism industry: A systematic literature review

**DOI:** 10.1016/j.heliyon.2024.e27256

**Published:** 2024-03-03

**Authors:** Jiru Zhang, Ivan Ka Wai Lai, Jose Weng Chou Wong

**Affiliations:** a Faculty of Hospitality and Tourism Management, Macau University of Science and Technology, Taipa, Macau; b Centre for Gaming and Tourism Studies, Macao Polytechnic University, Taipa, Macau

**Keywords:** Female tourism, Girlfriend getaway, Risk, Motivation, Sexuality

## Abstract

Female tourism is gaining momentum in the world. Thus, a review of female tourism is essential to explore future research directions. The paper discusses the evolution of female tourism research from the early 1980s to 2022 through different analytical methods. The 116 articles published in the list of Social Science Citation Index (SSCI) journals were collected. A systematic quantitative assessment of 116 articles was conducted using HistCite, and research themes were identified using VOSviewer. Qualitative content analysis was conducted to trace the growth of research, understand the past research development of each research theme, and identify research gaps for further research. The results show that current female tourism research can be divided into three research themes: motivation, risk, and sexuality. The motivations for young female travellers, solo female travellers, and middle-aged female travellers are discussed. In the atmosphere of gender equality, current female tourism research in risk and sexuality contains contradictory viewpoints with past studies as a result of changing times. A research agenda in four potential research areas is recommended. This review contributes to female tourism research by providing researchers with literature to guide and support further research.

## 1 Introduction

With the development of the economy, female travellers now account for more than half of the market share in both leisure and business travel [1]. Therefore, female tourism has become an important part of the tourism market. Female tourism has been documented in the travel literature since the mid-1980s [2]. In a general sense, it refers to the trip participants who were female and did not include a male [3]. In a narrow sense, female tourism defines a female vacation as a form of pleasure travel, primarily female travel with female friends or relatives [4]. 91% of female travellers go with companions, such as on a mother-daughter trip or one or several female friends, while the remainder travel alone [1]. The growth of the female tourism market has made the segmentation of the entire tourism industry more and more important [5,6], so female tourism has become one of the main topics of tourism studies. Therefore, understanding existing research is essential for researchers to track trends for further female tourism research.

Female tourism is a contentious subject that links between gender and tourism such as women's friendships, which may not work in males [7]. It is because women are more emotionally involved and dependent on friendships [8]. In addition, female tourism is both a kind of feminism and a practice of ideology-free of gender limitations [9]. such as shifting societal preconceptions, decompressing, making new acquaintances, or relaxing for the better [10]. Some female tourism studies have identified benefits of female tourism, including improved mental health, subjective well-being, and the use of brief vacations for self-transformation [11]. In addition to the benefits, researchers also investigated constraints on female travel, such as gender risk.

In examining different research contexts, some researchers reviewed existing female tourism research in their field. For example, Lenao and Basupi [12] reviewed the research on female empowerment in ecotourism. Yang et al. [13] reviewed the literature through a narrative method from a postcolonial feminist perspective and attempted to understand the impact of cultural identity and gender stereotypes on Asian female tourists. Their review found that most studies focused on segments of female travellers, such as nationality, beliefs, and perceptions of risks associated with females. The key to female tourism lies in the internal motivation and external feelings of female tourism, as well as the methodology and theoretical basis. Recently, Nisha and Cheung [14] conducted a systematic quantitative review of Muslim women's travel. They argued that perceptions and experiences of female Muslim tourism are primarily influenced by the religious and gender identities of female Muslim travellers. They also discussed the existing academic progress and research methods of Muslim female tourism research. Although the above reviews provide useful insight into female tourism in certain research areas, there is still a lack of a systematic review from a macro perspective to comprehensively summarize the results of the current literature and refine directions for future research. To advance intellectual female tourism knowledge, this study attempts to answer the following research question: "Based on current literature, what future directions we should have to conduct future research on female tourism?”

The objectives of this study include (1) tracing the growth of research into female tourism by classifying previous research on female tourism based on journal distribution, years of research publication, theoretical basis, and methodology; (2) using keywords to explore reveal research trends and comprehensive research topics of female tourism; and (3) proposing future research directions. There are several potential contributions to make this bibliometric study both theoretically and practically. Firstly, In the bibliometric study, the academic literature is systematically reviewed in this study, and all the themes, methodologies, viewpoints, levels of analysis (micro, macro, and general), and innovations discussed in the current literature are examined. Secondly, this study provides us with a chance to trace the origin and development process of this topic and extract a roadmap for future research on female tourism based on the analysis of highly cited articles.

## 2 Literature review

### 2.1 What is female tourism?

Female tourism is a phenomenon that combines female tourists of all ages travelling for leisure, including girlfriend getaway tourism [[15], [16], [17]]. From the late 1970s onwards, feminist studies began to emerge in leisure tourism research [2]. and since 1995, the feminist approach started to widespread and became a significant variable in human relations [18,19]. Nevertheless, female tourism only became a hotspot until the 1990s feminist empiricism focused on female legal rights [20]. Feminists are defined as establishing and owning equal political, and social rights for women [[21], [22], [23]]. Current feminist research argues that inequality has existed for a long time, and men have the dominant power in the relationships between men and women such as individual's social status and various opportunities in the lifetime [24]. Therefore, feminists addressed that the human rights and social status of females should be increased in order not to stereotype women as subordinates of men [25]. In the tourism field, knowing the background of the feminists helps us enrich our understanding of the complexities of female tourists in terms of their motivations, perceptions, and other travel behaviours [26].

### 2.2 Female tourism research

In the early stage, researchers took females as a whole group and the female tourism studies mainly focused on the general differences between female and male tourists (e.g. Ref. [27]). Recently, some studies attempted to examine this special group from different perspectives [22]. For instance, Jęczmyk et al. [28] examined how different female tourists define safety from male tourists during travel. In addition, some researchers would like to examine specific groups of female tourists, such as “girlfriend getaways” [15]. For instance, Wang et al. [29] examined the connection between escapism, happiness, luxury leisure, fulfilment, and well-being in girlfriend getaway tourism. Given that the development of female tourism is certain and ongoing, there is a need to conduct this systematic review, for researchers and practitioners to extract future trends of female tourism from the development of female tourism in the past few decades.

## 3 Methodology

To gain an insightful understanding of the existing literature on female tourism and extract future trends, this study follows the standard literature review methods employed by the existing systematic reviews [[30], [31], [32]]. Specifically, the systematic review process undertaken in this study was adapted mainly from Pickering and Byrne [33] and Basu et al. [34].

Different from other qualitative-based reviews that apply a textual-based analysis, this study applied a mixed method of quantitative and qualitative analysis. Specifically, this study combines co-citation analysis, cartography analysis, and a qualitative content analysis. The flow chart is also adapted from PRISMA and is used to understand the process of literature search and detection and determine future research directions. This research consists of three main steps, including data collection, data analysis procedures, and content analysis.

### 3.1 Data collection

**Fig. 1:**
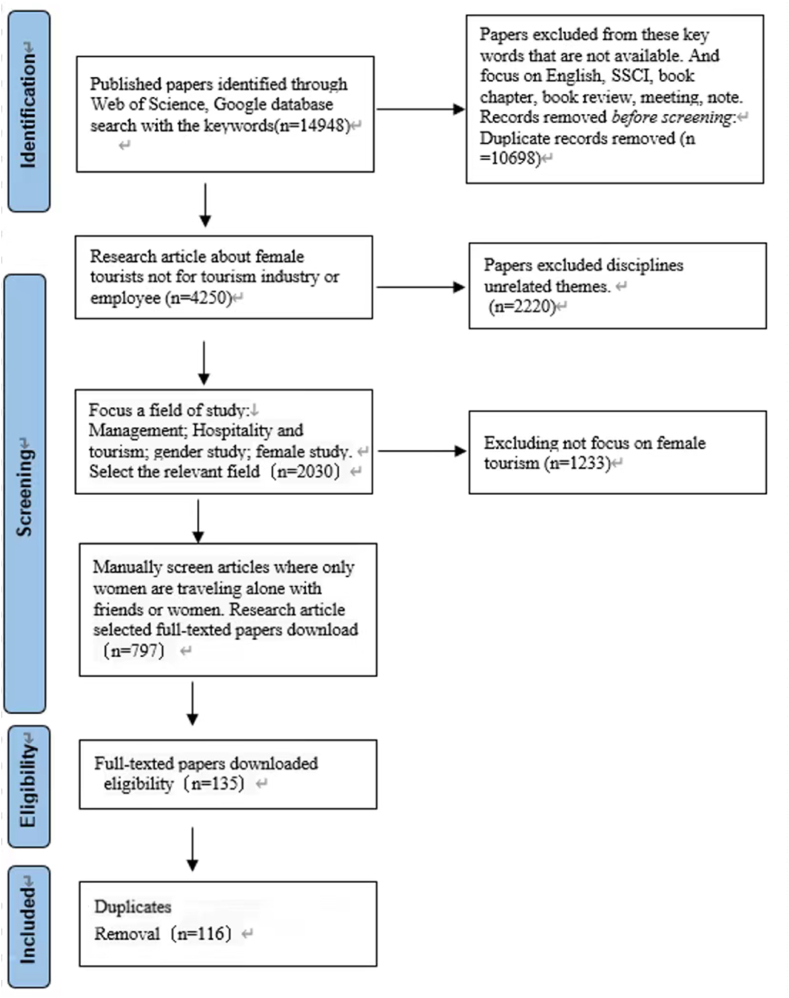
PRISMA research process. (Source: the authors' own elaboration).

The first stage of the review process involved collecting and organizing existing literature. To consolidate the state of female tourism research, articles on female tourism were the objects of the study for this review. The data on the Web of Science (WoS) were collected in April 2022. Only the database source in the list of Social Science Citation Index (SSCI) was included by searching the keywords: “female tourism”, “female travel”, “girlfriend getaway”, “women tourism”, and “women travel”. 797 articles were collected. Then, the abstract and content of the articles were reviewed, and the non-conformities were removed. Articles that did not belong to the scope of female tourism were removed to ensure all articles included were about female tourism studies. Finally, the manual deletion of irrelevant articles brought the dataset down to 116 articles. The PRISMA flowchart of this study is shown in Fig. 1.

#### 3.1.1 Data analysis procedures

In this research, a systematic quantitative and qualitative assessment of the literature on 116 female tourism publications relevant to tourism studies was undertaken. On the quantitative assessment, two types of bibliometric analysis were used (co-citation using HistCite, and bibliographic coupling and cartography analysis using VOSviewer). Bibliometric analysis systematically classified published articles in terms of publication year, author names, institutions and countries, author numbers, collaboration type, nature of the article, and different themes [35,36]. Research themes were identified using VOSviewer.

### 3.2 Content analysis

Qualitative content analysis was conducted by reviewing 116 articles on female tourism to understand the past research development of each research theme and identify research gaps for further research.

### 3.3 Findings

#### 3.3.1 General information

**Fig. 2:**
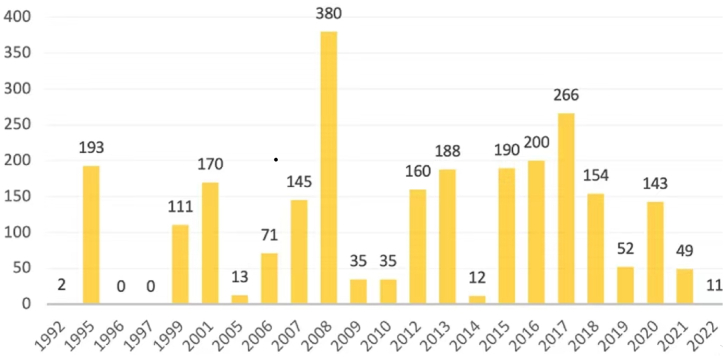
Times cited by year.

The citation graph (Fig. 2) shows the number of citations by year. It indicates that female tourism continues to appear in the literature. It discovers that the earliest high number of citations was in 1995 (193 citations), the highest number of citations was in 2008 (380 citations), and the next wave of high citations was from 2015 to 2017 (190, 200, and 266 citations). Between 2012 and 2018, many notable scholars have written articles in this field.

#### 3.3.2 Analysis of the total global citation score

These 116 articles were published in a total of hospitality and tourism journals. According to the total global citation score (TGCS), the most important journals were evaluated using the list of top 10 journals with the help of HistCite, as shown in Table 1. *Annals of Tourism Research* and *Tourism Management* were the two journals which published the most papers on female tourism (15 papers). *Current Issues in Tourism* was followed next, which published 12 papers. Then, *Gender Place and Culture* followed, which published 9 papers.

**Table 1:** Number of articles published in hospitality and tourism journals.

	Journal	Items	TGCS	Author	Items	TGCS
1	Annals of Tourism Research	15	576	Khoo-Lattimore	12	240
2	Tourism Management	15	665	Berdychevsky	10	234
3	Current Issues in Tourism	12	358	Gibson	8	144
4	Gender Place and Culture	9	63	Prayag	5	82
5	International Journal of Tourism Research	6	61	Yang	5	123
6	Tourism Management Perspectives	6	39	Brown	4	105
7	International Journal of Hospitality Management	5	45	Poria	4	106
8	Journal Of Sustainable Tourism	4	144	Small	4	157
9	Leisure Studies	4	103	Arcodia	3	106
10	Journal Of Gender Studies	3	3	Heimtun	3	52

The most influential authors were ranked according to the total global citation score (TGCS). The first was Khoo-Lattimore C, with a TGCS of 240, and the second was Berdychevsky, with a TGCS of 234. Small J was the third, with a score of 157. The following authors were Gibson (TGCS = 144) and Yang (TGCS = 123).

In terms of countries/regions and institutions, a total of 125 institutions from 31 countries/regions co-authored 116 publications. The top 10 countries/regions and institutions ranked by number of publications are shown in Table 2. The top 5 countries/regions are distributed in Australia (n = 31), the USA (n = 29), the UK (n = 22), China (n = 12), and New Zealand (n = 8). The top 5 institutions are Griffith University, University of Florida, University of Illinois, Ben Gurion University Negev, and Bournemouth University.

**Table 2:** The top 10 countries and institutions involved.

	Country	Count	Institution	Count
1	Australia	31	Griffith Univ	15
2	USA	29	Univ Florida	12
3	UK	22	Univ Illinois	8
4	Peoples R China	12	Ben Gurion Univ Negev	5
5	New Zealand	8	Bournemouth Univ	5
6	Malaysia	7	Hong Kong Polytech Univ	5
7	Unknown	7	Univ Canterbury	5
8	Israel	6	Univ Technol Sydney	4
9	Spain	4	Finnmark Univ Coll	3
10	Canada	3	Taylors Univ	3

#### 3.3.3 Citation mapping

**Fig. 3:**
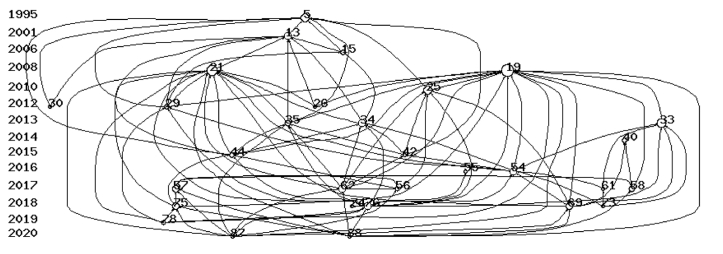
Citation map of the 30 most cited articles produced by HistCite.

HistCite common citation analysis is shown in Fig. 3. Based on the TGCS, the 30 most cited articles were identified. Fig. 3 shows the 30 articles (nodes) and 101 links (relationships between articles). These articles demonstrate a powerful integrated citation mapping, indicating that prominent academic scholars have given high attention to published works and have cited them in their research papers. HistCite citation mapping exhibits a clear distinction of one seminal research article, presented as the centre of many connections (represented as number 5 in Fig. 3). The full list of the 30 most cited articles is shown in Table 3.

**Table 3:** The list of 30 most cited articles.

Number	Node	Author	Year	Title	Journal
1	5	Pruitt, D., & LaFont	1995	For love and money: romance tourism in Jamaica.	Annals of tourism research
2	13	Herold, Garcia, & DeMoya	2001	Female tourists and beach boys: romance or sex tourism?	Annals of tourism Research
3	15	Taylor	2006	Female sex tourism: a contradiction in terms?	Feminist review
4	19	Wilson & Little	2008	The solo female travel experience: Exploring the ‘geography of women's fear’	Current Issues in Tourism
5	21	Jordan & Aitchison	2008	Tourism and the sexualisation of the gaze: Solo female tourists' experiences of gendered power, surveillance and embodiment.	Leisure Studies
6	25	McNamara & Prideaux	2010	A typology of solo independent women travellers.	International Journal of Tourism Research
7	26	Heimtun & Abelsen	2012	The tourist experience and bonding.	Current Issues in Tourism
8	29	Heimtun	2012	The friend, the loner and the independent traveller: Norwegian midlife single women's social identities when on holiday.	Gender, Place & Culture
9	30	Weichselbaumer	2012	Sex, romance and the carnivalesque between female tourists and Caribbean men.	Tourism Management
10	33	Berdychevsky, Gibson, & Bell	2013	Girlfriend getaways and women's well-being.	Journal of Leisure Research
11	34	Berdychevsky, Poria, & Uriely	2013	Sexual behavior in women's tourist experiences: Motivations, behaviors, and meanings.	Tourism Management
12	35	Berdychevsky, Gibson, & Poria	2013	Women's sexual behavior in tourism: Loosening the bridle.	Annals of Tourism Research
13	40	Guo	2014	Chinese women and travel: historical and contemporary experiences.	Annals of Tourism Research
14	42	Berdychevsky, Gibson, & Poria	2015	Inversions of sexual roles in women's tourist experiences: Mind, body, and language in sexual behaviour.	Leisure Studies
15	44	Berdychevsky, & Gibson	2015	Phenomenology of young women's sexual risk-taking in tourism.	Tourism Management
16	54	Berdychevsky, Gibson, & Bell	2016	“Girlfriend getaway” as a contested term: Discourse analysis	Tourism Management
17	55	Gao & Kerstetter	2016	Using an intersectionality perspective to uncover older Chinese female's perceived travel constraints and negotiation strategies.	Tourism Management
18	56	Khan, Chelliah., & Ahmed	2017	Factors influencing destination image and visit intention among young women travellers: Role of travel motivation, perceived risks, and travel constraints.	Asia Pacific Journal of Tourism Research
19	57	Zhang, & Hitchcock	2017	The Chinese female tourist gaze: A netnography of young women's blogs on Macao.	Current Issues in Tourism
20	58	Yang,Khoo-Lattimore, & Arcodia	2017	A narrative review of Asian female travellers: Looking into the future through the past.	Current Issues in Tourism
21	61	Durko, & Stone	2017	Even lovers need a holiday: Women's reflections of travel without their partners.	Tourism Management Perspectives
22	62	Brown, & Osman	2017	The female tourist experience in Egypt as an Islamic destination.	Annals of Tourism Research
23	69	Seow & Brown	2020	The solo female Asian tourist.	Current Issues in Asian Tourism
24	73	Mirehie, Gibson, Khoo-Lattimore, & Prayag	2018	An exploratory study of hospitality needs and preferences of US Girlfriend Getaways.	Journal of Hospitality Marketing & Management
25	74	Pritchard	2018	Predicting the next decade of tourism gender research.	Tourism Management Perspectives
26	75	Yang, Khoo-Lattimore, & Arcodia	2018	Constructing space and self through risk taking: A case of Asian solo female travelers.	Journal of Travel Research
27	76	Yang, Khoo-Lattimore, & Arcodia	2018	Power and empowerment: How Asian solo female travellers perceive and negotiate risks.	Tourism Management
28	78	Yang, Yang, & Khoo-Lattimore	2019	The meanings of solo travel for Asian women.	Tourism Review
29	87	Osman, Brown, & Phung	2020	The travel motivations and experiences of female Vietnamese solo travellers.	Tourist Studies
30	88	Su, & Wu	2022	The dark side of solo female travel: Negative encounters with male strangers.	Innovation and Impact of Sex as Leisure in Research and Practice

As shown in the figure above, Pruitt and LaFont's study [37] (No. 5) was the first to lead the investigation of female tourism. Their study provides a unique perspective on gender transition by examining the relationships between foreign female tourists and local men, having women try out masculine behaviours. With the rise of mass tourism, women are breaking free from the confines of their own society and constantly experimenting with new gender behaviours while travelling. After that, Herold et al. [38] (No. 13) and Taylor [24] (No. 15) described female tourism from the perspective of female sex tourism. Herold et al. [38] found that the relationship between female tourists and local beach boys is defined as involving sexual tourism or romantic tourism. Their study found that female tourists are more inclined towards romance, while men are more inclined towards sex. Taylor [24] explored the underbelly of gender rights, sexual exploitation, and theoretical knowledge at the time, and understood the boundaries between commercial and non-commercial sex through understanding the different standards of sexuality held by male and female tourists. Subsequently, some studies like Wilson and Little [39] (No.21) and Jordan and Aitchison [5] (No.19) investigated female tourism in another way. Their research was from the perspective of women travelling alone to understand the risks and constraints women face. During that period, there was a lot of research on solo female travel that aimed to understand the solo female tourism experience through the lens of the male gaze.

Later studies examined female tourism from both a male and social perspective. For example, research on women's inherent social responsibilities, such as family care and child-rearing constraints, and tourism well-being [40]. Since then, research on female tourism has begun, and from the 2010s to the present, most of the research has been conducted from the perspective of sex and risk (e.g. No. 42). Another stream of studies was on Asian women and Muslim women, which provided contemporary academic analysis and interpretation of female travellers and tourism phenomena from a perspective of tourism, such as revealing the multifaceted elements of female travel [41]. The diversity of travel experiences and perspectives, especially from women from other cultures, highlights the importance of influencing regional culture and inspires industry players to pay more attention to market segmentation among key travel groups.

### 3.4 Research themes

**Fig. 4:**
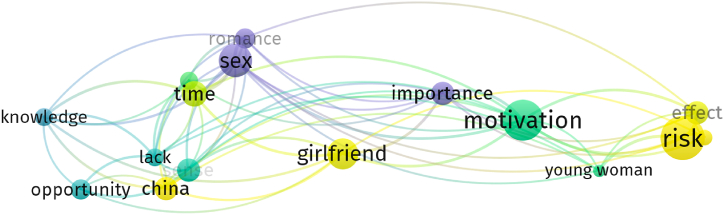
Research themes in female tourism.

After the preliminary analysis by HistCite, 116 articles were imported into VOSviewer to extract a bibliographic coupling and cartography analysis for female tourism. Fig. 4 shows that there are three research themes of female tourism, including motivation, risk, and sexuality.

#### **3.4.1** Motivation

Among 116 articles, 47 articles belonged to the ‘motivation’ research theme. The motivations for women to travel include the following: challenge themselves, socialize, meet new people, extend beyond personal, comfort zones, develop a feeling of autonomy and independence, self-empowerment, and extend themselves. This theme can be divided into 3 sub-themes according to their age groups and travel patterns.

(1) Young female travellers

Young female travellers are motivated by discovering landscapes and sightseeing, natural environment and culture, rest and relaxation, meeting new acquaintances, and visiting family and friends which influence their destination choice [32]. Young female travellers spend quality time with their friends, break the routine, or rejoice at the beach or in the city, and they are interested in life-changing experiences, identities, and self-empowerment [42]. The motivations for young women to travel include relieving tension, escaping routines, daily constraints and social norms, social status, and caring ethics.

(2) Solo female travellers

The motivations for solo female travellers are seeking self-identification, self-empowerment, and increased self-confidence and self-esteem. The solo female travel motivation for Asian women also includes a transformative experience [43]. There is also a concept of transition in the motivation of young solo female travellers, such as transitioning from a student to a career woman, or single travel before marriage [44]. In addition, due to these stereotypes from the outside world [1], solo female travellers are more willing to travel to break the constraints through travel and seek connections with global citizens [43].

(3) Middle-aged and senior female travellers

Middle-aged women feel the fading of youth, so they want to 'feel young again' before they become older, while for widowed women, travelling with best friends can make up for their inner weakness. The stigma of being a middle-aged single woman in a family or couples travel environment is minimized by creating connections and independence, which is exactly what women desire in travel, especially since the more children women have, the less likely they travel [45].

The studies on the motivations of female tourists highlighted the differences in the motivations of women of different ages. For instance, solo female travellers can take part in different types of activities that interest them [46]. On the other hand, middle-aged and older female travellers prefer to experience life with their friends or female partners. However, there are some common motivations among different groups of female travellers.

### 3.5 Risk

Among 116 articles, 34 articles belong to the ‘risk’ research theme. Many previous studies have shown that female travellers face risks when engaging in tourism activities than male travellers [47], such as sexual violence [48], gender bias [1], and limited access [49] because of the gender. Fernández, Pena-Boquete, and Pereira [50] stated “the greater risk of gender discrimination” to describe female tourism. This theme can be divided into 2 sub-themes according to different perceived risks in gender and females themselves.

(1) Differences in perceived travel risks by gender

Some studies argued that women and men may perceive risk differently because women face some risks from men, such as the risk of sexual violence [51]. In addition to understanding gendered risk perceptions, other scholars have shown that societal acceptance of risk-taking behaviour differs by gender. Risk is often equated with masculinity, but when a girl and risk are combined the word can become very negative. Jordan and Aitchison [5] observed the self-monitoring tendencies of female tourists. For women, risks are everywhere, such as geographic risks. Europe, the Middle East, and East Asia are all considered dangerous places, such as the cause of wars and local disrespect for women [51]. Even women travel in safe places with risk management, such as not going out at night because they know screaming is useless because no one will come to save them [39]; or dressing conservatively and hiding their femininity, sometimes they also add some tools to reduce their own wind direction, such as smoking on the road at night, or wearing a fake wedding ring [52]. Brown and Osman [18] argued that men's preoccupation with sexiness severely limits women's ability to enjoy travel because they fear for their comfort and safety. Although previous studies have highlighted the gender risks of women travelling, in an atmosphere of gender equality, recent research appears to be changing this view [17].

(2) Differences in perceived travel risks by female themselves

While female travellers face higher risks and restrictions, participating in risky activities can allow female travellers to feel independent and self-affirming [1]. Multiple studies have shown that risk perceptions and constraints come primarily from tourist destinations [53]. With risks come risks, but some women don't necessarily agree. But women also perceive the risks they encounter as good things, and overcoming fears and dealing with their troubles during travel will give women a stronger sense of agency, liberation, independence, and power [43]. Then the risk ameliorating factors for women are mainly their own travel attitude and travel experience. Women who travel frequently have lower risk perceptions and are more relaxed during travel, while tourists who are first-time travellers have much higher risk perceptions. In addition, adventurous tourists are more willing to travel to places that involve a certain amount of risk. Weatherby and Vidon [54] also found that more and more women are travelling to remote areas to demonstrate their desire to conquer, to prove their power to themselves and others, to break down societal stereotypes about women, and to redefine femininity. It is also a positive form of women's resistance to patriarchal society and patriarchal expectations through travel.

#### **3.5.1** Sexuality

Among 116 articles, 35 articles belong to the research theme of ‘sexuality’. In this theme, there are more studies on female sexuality and comparing the sexes, such as the studies on female tourists with local men under the male gaze and studies on women being socially addicted to travel and socialising than men [39]. This theme can be divided into 2 sub-themes according to conflict situations in women.

(1) Challenges to gender

Under the trends of pursuing human equality [55,56], female travellers also do things that only men do in the public eye to achieve gender equality, as Pilcher [57] described women's experiences in male strip show venues as a sexy and special romance experience, with ways to transcend gender roles, release constraints, and express sexuality positively. Western women engage in nominally ‘masculine’ behaviours while travelling, such as building relationships and becoming sexual partners [58]. Their purpose is to experience it by adopting a ‘masculine’ identity. Researchers see this as a challenge to gender and racial inequality as well as a dissatisfaction with reality. Female travellers will look for romance in tourism, rather than pursuing sex like men, which is the essence of female travellers [59]. In terms of sexual experience, privacy is also an essential element for women in the hospitality industry as women travel in an anonymous environment, travel is an opportunity for casual sex without reputational impact [8].

(2) Women's inferiority

Women also have different situations from the perspective of their own gender. While women have made some advances in travelling, their negative thoughts about their appearance limit their involvement or lessen their enjoyment, since there will be fewer activities requiring bathing suits due to aesthetic issues [60]. Limited research on female appearance during travel for the lived experience of travel confirms the importance of women's appearance during the holidays, with women feeling positive about their bodies when they meet their ideal body shape. When they fail to meet the standard, they feel negative about their body shape. They pursue that their bodies and takeaways must be at their best while travelling [61].

## 4 Future research agenda

### 4.1 Motivation

In terms of motivations, the studies of women's motivations are dominated by the tourism situation of the destinations, further studies may be performed on the perceptual motivations of herself, such as a sense of connection between girlfriends and families (mother and daughter). Previous studies on Western women may have changed owing to the passage of time and the development of the feminist movement.

Time and opportunities for female tourists to travel are also important to women, as women need to take care of children and family outside of work, which in many countries is a woman's domestic responsibility. Therefore, time is also a major factor that women consider when travelling. In addition, residents and foreign tourists have been exposed to different cultures and educations, so there may be significant differences in behaviour or dressing. Sometimes female tourists gradually enter the entire market and behave differently from local women, such as drinking and wearing swimsuits, which are different from local women. Temperaments may be quite different, with local women wearing veils and engaging in activities such as farming [62]. Feminist research is particularly numerous in postcolonial feminist theory, which attempts to advance gendered research, such as male/female, colonizer/colonized, and hegemony/subordination [63]. In the process, postcolonial feminism paves the way for a more nuanced understanding of feminism.

With the development of smart technology, some intelligent tourism services and mobile APPs are specially designed for female travel. Therefore, how these technologies motivate women to travel more frequently is worth studying. In addition, studies on postmodern tourists are increasing, so it is also possible to study the motivations of female postmodern tourists. Female travellers in different regions and religions may aim for different purposes, so future studies can also be used to compare their travel motivations.

### 4.2 Risk

Some current studies focus on the area of risk. A growing body of research links risk to sex, as women are generally at higher risk of sexual violence than men. However, as women become independent, some past risks may no longer only apply to women or cause harm to women [64]. For example, women's risk perception will be different on women-friendly trains in Japan. In addition, past research has been relatively one-sided, and sexual violence or unwanted sexual behaviour may also occur in men. In addition, the sexual behaviour of female tourists also needs to be based on their own feelings and understand the perceived value and experience of female tourists themselves, rather than from the gaze of men and society. Also, many studies were conducted from the perspective of the environment, while neglecting the pros and cons of new technologies, such as positioning systems or AI, or employing ICT technology to lessen women's risks, such as Didi's one-click alert feature [65]. When women participate in night tourism, the use of smart technology may make them feel comfortable in terms of risk management due to its alert function. As more and more women participate in self-driving tourism, female drivers may be concerned about their safety and try to reduce the risk of accidents. Also, a great number of research on women's risks are associated with sex and the negative aspects of risk, and it is also possible to examine the positive aspects of women's travel risks.

### 4.3 Sexuality

In terms of sexuality, a large portion of current research focused on the female population as a whole, ignoring demographic differences. Since there are many stages in a person's life (such as single, married, graduated), current studies rarely consider subdividing the stages of people's life, so future research could be studied from the perspective of demographic classification, such as age, marital status (whether to have children, how many children to have) conduct a comparative analysis, or to understand the sexual of women with different educational backgrounds. On the other hand, most sexual currently refers to sexual behaviour, but less research begins with women's own constraints, such as women's self-objectification, becoming fat, or choosing not to travel because they do not have beautiful clothes [66]. Some environmental impacts on women's travel restrictions should also be considered, such as prohibiting women from travelling due to cold or sun exposure. It is suggested that in the future, it can be studied from other perspectives besides sex.

### 4.4 Others

In addition, there are fewer studies investigating the consumption behaviour of various types of female tourism consumers based on age and marital status, and there are few studies related to consumption motivations [31]. Without support from empirical research, we may not know whether women's travel motivations differ from men's. Also, current studies mostly focused on how the attributes of the tourism destinations attract females to visit, but few studies have examined how different forms of travel (e.g., crucial tourism or adventure tourism) influence female travel decisions. Travel forms such as medical beauty tourism mainly target the female tourism market. In terms of tourism promotion content, current studies showed that women are more likely than men to read comments with pictures and texts [67]. Given that new forms of social media channels are emerging [65,68], further research can be conducted from a new technological perspective to see how new social media channels influence or attract female travellers.

## 5 Conclusions and implications

### 5.1 Summary of research and findings

This gender research adopts a feminist theoretical perspective to conduct contemporary academic analysis and interpretation of female travellers and tourism phenomena. This study conducted a systematic review of 116 articles published from 1992 to 2022, revealing the complexity of female tourism. While gaining in-depth information and insights, this study also emphasizes the need for academics to critically and multifacetedly articulate the notion of female tourism. Given that tourism experiences may vary greatly between different groups [69,70], the tourism experiences of women from other cultures or regions may have great differences, and we should pay closer attention to the market segmentation of the important tourism groups.

### 5.2 Theoretical implications

The contribution of this study is valuable by providing a systematic review of female tourism. Firstly, this study applied a quantitative method to trace the growth of research into female tourism. Secondly, by using the Histcite and Vosviewer, three main themes (motivation, risk, and sexuality) are extracted. Thirdly, through the content analysis of the articles, this study provided a clearer picture of the previous studies and helped researchers identify core areas and key findings in the field of female tourism. Finally, this study also developed a further research agenda that covers four domains, including motivation, risk, sexuality, and others. Future studies could be conducted on different themes where researchers may plan to consider non-gender (only from the female perspective) or gender (comparison between female and male) in these research domains.

### 5.3 Practical implications

Research provides not only academic implications but also practical implications. As a destination manager or practitioner, understanding the themes of female tourism may allow them to better understand this specific group and create tourism products that are better suited to their needs and desires. In particular, women's risk perception and the motivation for girlfriend getaways, this study provides product design ideas and product optimization for tourism-related enterprises, such as designing women-only rooms, women-only hotel butlers, and so on. Therefore, this study provides a comprehensive overview of the current status of female tourism, provides inspiration and reference for practitioners, and provides important experimental contributions to the tourism and hospitality industry.

### 5.4 Study limitations

This study has several limitations that need to be improved. Firstly, the scope of analysis only includes the analysis of papers published in SSCI journals, and in the future, more reference research notes, books, etc. will be needed. Then the systematic literature focused on only the Web of Science database, some studies from other databases (such as Scopus) were not included in this study. Some recent papers that have not been highly cited may not make it into the top thirty citations; some follow-up studies are recommended to track those recent articles. Secondly, given the qualitative nature of content analysis in this study, the generalization of the findings needs to be interpreted carefully.

### 5.5 Future research

As mentioned above, the scope of analysis only included analysis of journal articles, so future studies are encouraged to include other databases and sources to enrich the generalizability of this study. In addition, following the findings related to female travellers in this study, further research may be extended to some specific groups of female travellers, such as postmodern female tourists, or by considering other demographic characteristics (such as age group, marital status, education, race and so on) to provide in-depth insights.

## Funding statement

The authors declare that no funds and grants were received for the preparation of the manuscript.

## Data availability statement

This is a systematic review and therefore all data are included in the manuscript.

## Ethics statement

An ethics statement is not applicable because this study is based exclusively on published literature.

## CRediT authorship contribution statement

**Jiru Zhang:** Writing – review & editing, Writing – original draft, Formal analysis, Data curation, Conceptualization. **Ivan Ka Wai Lai:** Writing – review & editing, Writing – original draft, Conceptualization. **Jose Weng Chou Wong:** Writing – review & editing, Writing – original draft, Supervision, Data curation, Conceptualization.

## Declaration of competing interest

The authors declare that they have no known competing financial interests or personal relationships that could have appeared to influence the work reported in this paper.

## References

[1] Khan M.J., Chelliah S., Khan F., Amin S. (2019). Perceived risks, travel constraints and visit intention of young women travelers: the moderating role of travel motivation. Tourism Rev..

[2] Aitchison C.C. (2005). Feminist and gender perspectives in tourism studies: the social-cultural nexus of critical and cultural theories. Tour. Stud..

[3] Khoo-Lattimore C., Prayag G. (2015). The girlfriend getaway market: segmenting accommodation and service preferences. Int. J. Hospit. Manag..

[4] Doran A., Schofield P., Low T. (2020). Women's mountaineering: accessing participation benefits through constraint negotiation strategies. Leisure Stud..

[5] Jordan F., Aitchison C. (2008). Tourism and the sexualisation of the gaze: solo female tourists' experiences of gendered power, surveillance and embodiment. Leisure Stud..

[6] Junek O., Binney W., Winn S. (2006). All-female travel: what do women really want?. Tourism Int. Interdiscipl. J..

[7] Heimtun B., Jordan F. (2011). Wish YOU Weren't here!’: interpersonal conflicts and the touristic experiences of Norwegian and British women travelling with friends. Tour. Stud..

[8] Berdychevsky L., Poria Y., Uriely N. (2013). Sexual behavior in women's tourist experiences: motivations, behaviors, and meanings. Tourism Manag..

[9] Ballaster R., Beetham M., Frazer E., Hebron S. (1991).

[10] Berdychevsky L., Gibson H.J. (2015). Phenomenology of young women's sexual risk-taking in tourism. Tourism Manag..

[11] Berdychevsky L., Poria Y., Uriely N. (2013). Hospitality accommodations and women's consensual sex. Int. J. Hospit. Manag..

[12] Lenao M., Basupi B. (2016). Ecotourism development and female empowerment in Botswana: a review. Tourism Manag. Perspect..

[13] Yang E.C.L., Khoo-Lattimore C., Arcodia C. (2017). A narrative review of Asian female travellers: looking into the future through the past. Curr. Issues Tourism.

[14] Nisha F., Cheung C. (2022). Locating Muslimah in the travel and tourism research. Tourism Manag. Perspect..

[15] Berdychevsky L., Gibson H.J., Bell H.L. (2016). “Girlfriend getaway” as a contested term: discourse analysis. Tourism Manag..

[16] Ćurčić N., Zakić L., Galantić M. (2009). Segmentation of tourist market-Women as consumers. Geographica Temisiensis.

[17] Wang X., Lai I.K.W., Wang K. (2023). Do young women travellers really consider the risk of sexual harassment during night travel? evening travel vs midnight trave. Tourism Rev..

[18] Brown L., Osman H. (2017). The female tourist experience in egypt as an islamic destination. Ann. Tourism Res..

[19] Eger C., Munar A.M., Hsu C. (2023). Gender and Tourism Sustainability.

[20] Pritchard A. (2018).

[21] Hooks B. (2000).

[22] Long F., Aziz N.A. (2022). Travel abroad for face gaining or face saving? a comparison between Chinese gen Y male and female tourists in a context of Chinese culture. J. Int. Consum. Market..

[23] Mohajan H. (2022).

[24] Taylor J.S. (2006). Female sex tourism: a contradiction in terms?. Fem. Rev..

[25] Almela M.S., Calvet N.A. (2021). Volunteer tourism and gender: a feminist research agenda. Tourism Hospit. Res..

[26] Schneider M.C., Bos A.L. (2019). The application of social role theory to the study of gender in politics. Polit. Psychol..

[27] Kousis M. (1989). Tourism and the family in a rural Cretan community. Ann. Tourism Res..

[28] Jęczmyk A., Uglis J., Zawadka J., Pietrzak-Zawadka J., Wojcieszak-Zbierska M.M., Kozera-Kowalska M. (2023). Impact of COVID-19 pandemic on tourist travel risk perception and travel behaviour: a case study of Poland”. Int. J. Environ. Res. Publ. Health.

[29] Wang X., Lai I.K.W., Wang X. (2023). The influence of girlfriend getaway luxury travel experiences on women's subjective well-being through travel satisfaction: a case study in Macau. J. Hospit. Tourism Manag..

[30] Bernard S., Rahman I., McGehee N.G. (2022). Breaking barriers for Bangladeshi female solo travelers. Tourism Manag. Perspect..

[31] Li C.S., Zhang C.X., Chen X., Wu M.S.S. (2021). Luxury shopping tourism: views from Chinese post-1990s female tourists. Tourism Rev..

[32] McNamara K.E., Prideaux B. (2010). A typology of solo independent women travellers. Int. J. Tourism Res..

[33] Pickering C., Byrne J. (2014). “The benefits of publishing systematic quantitative literature reviews for PhD candidates and other early-career researchers”. High Educ. Res. Dev..

[34] Basu R., Lim W.M., Kumar A., Kumar S. (2023).

[35] Hjalager A.-M. (2010). A review of innovation research in tourism. Tourism Manag..

[36] Shah S.H.H., Lei S., Ali M., Doronin D., Hussain S.T. (2019). Prosumption: bibliometric analysis using HistCite and VOSviewer. Kybernetes.

[37] Pruitt D., Lafont S. (1995). For love and money: romance tourism in Jamaica. Ann. Tourism Res..

[38] Herold E., Garcia R., Demoya T. (2001). Female tourists and beach boys: romance or sex tourism?. Ann. Tourism Res..

[39] Wilson E., Little D.E. (2008). The solo female travel experience: exploring the ‘geography of women's fear. Curr. Issues Tourism.

[40] Berdychevsky L., Gibson H.J., Bell H.L. (2013). Girlfriend getaways and women's well-being. J. Leisure Res..

[41] Yang E.C.L., Khoo-Lattimore C., Arcodia C. (2017). A systematic literature review of risk and gender research in tourism. Tourism Manag..

[42] Mirehie M., Gibson H.J., Khoo-Lattimore C., Prayag G. (2018). An exploratory study of hospitality needs and preferences of U.S. Girlfriend Getaways. J. Hospit. Market. Manag..

[43] Wilson E., Harris C. (2006). Meaningful travel: women, independent travel and the search for self and meaning. Tourism Int. Interdiscipl. J..

[44] Terziyska I. (2021). Solo female travellers: the underlying motivation. Gender and Tourism.

[45] Rosenbloom S. (2004).

[46] Wang S., Lai I.K.W., Wong J.W.C. (2023). The impact of pluralistic values on postmodern tourists' behavioural intention towards renovated heritage sites. Tourism Manag. Perspect..

[47] Wilson E., Little D.E. (2005). A “relative escape”? The impact of constraints on women who travel solo”. Tourism Rev. Int..

[48] Thomas T.K., Mura P. (2019). The ‘normality of unsafety’-foreign solo female travellers in India. Tour. Recreat. Res..

[49] Seow D., Brown L. (2018). The solo female Asian tourist. Curr. Issues Tourism.

[50] Fernández M., Pena-Boquete Y., Pereira X. (2009). Labor conditions in the Spanish hotels and restaurants industry. Tourism Anal..

[51] Christensen T.J. (1999). China, the US-Japan alliance, and the security dilemma in East Asia. Int. Secur..

[52] Yang E.C.L., Khoo-lattimore C., Arcodia C. (2018). Constructing space and self through risk taking: a case of Asian solo female travelers. J. Trav. Res..

[53] Su C.-P., Wu T.-C. (2020). The dark side of solo female travel: negative encounters with male strangers. Leisure Sci..

[54] Weatherby T.G., Vidon E.S. (2018). Delegitimizing wilderness as the man cave: the role of social media in female wilderness empowerment. Tour. Stud..

[55] Lai I.K.W., Wong J.W.C. (2022). Comparing the effects of tourists' perceptions of residents' emotional solidarity and tourists' emotional solidarity on trip satisfaction and word-of-mouth intentions. J. Trav. Res..

[56] Lai I.K.W., Wong J.W.C., Hitchcock M. (2022). A study of how LGBTQ tourists' perceptions of residents' feelings about them affect their revisit intentions: an emotional solidarity perspective. J. Sustain. Tourism.

[57] Pilcher K.E.M. (2013). A ‘sexy space’for women? Heterosexual women's experiences of a male strip show venue. Sexualities, Spaces and Leisure Studies.

[58] Jacobs J. (2009).

[59] Pilcher K. (2019). Women and sex tourism landscapes. Fem. Theor..

[60] Small J. (2022). The sustainability of gender norms: women over 30 and their physical appearance on holiday. J. Sustain. Tourism.

[61] Weichselbaumer D. (2012). Sex, romance and the carnivalesque between female tourists and Caribbean men. Tourism Manag..

[62] Tong J.K.-C., Turner B.S. (2016). The Sociology of Islam.

[63] Singh N. (2015). Unveiling the veiled face: imtiaz dharker's ‘purdah’and the colonial stereotype. International journal of English language. Literature and Humanities.

[64] Karagöz D., Işık C., Dogru T., Zhang L. (2021). Solo female travel risks, anxiety and travel intentions: Examining the moderating role of online psychological-social support”. Curr. Issues Tourism.

[65] Ng K.S.P., Wong J.W.C., Xie D., Zhu J. (2023). From the attributes of smart tourism technologies to loyalty and WOM via user satisfaction: the moderating role of switching costs. Kybernetes.

[66] Prichard I., McLachlan A.C., Lavis T., Tiggemann M. (2018). The impact of different forms of fitspiration imagery on body image, mood, and self-objectification among young women. Sex. Roles.

[67] Taipale S. (2014). The affordances of reading/writing on paper and digitally in Finland. Telematics Inf..

[68] Zhou X., Wong J.W.C., Xie D., Liang R., Huang L. (2023). What does the audience care? The effects of travel vlog information quality on travel intention. Total Qual. Manag. Bus. Excel..

[69] Wong J.W.C., Lai I.K.W., Tao Z. (2019). Memorable ethnic minority tourism experiences in China: a case study of Guangxi Zhuang Zu. J. Tourism Cult. Change.

[70] Wong J.W.C., Lai I.K.W., Tao Z. (2020). Sharing memorable tourism experiences on mobile social media and how it influences further travel decisions. Curr. Issues Tourism.

